# Short-term effect of a moderate-potency topical corticosteroid on epidermal biophysical parameters in patients with mild-to-moderate atopic dermatitis: A randomised controlled study

**DOI:** 10.51866/oa.611

**Published:** 2024-08-18

**Authors:** Zainal Abdullah Zainal Adlishah, Adawiyah Jamil

**Affiliations:** 1 MBBChBAO, MMed (Int. Med.), AdvMDerm, Department of Medicine, Faculty of Medicine, University Kebangsaan Malaysia, Cheras, Kuala Lumpur, Malaysia. Email: adawiyahjamil@ukm.edu.my; 2 MBBCh, Doc.Int.Med., Department of Medicine, Faculty of Medicine, University Kebangsaan Malaysia, Cheras, Kuala Lumpur, Malaysia.

**Keywords:** Atopic dermatitis, Eczema, Steroid, Water loss

## Abstract

**Introduction:**

Skin barrier dysfunction is an important component of atopic dermatitis (AD) pathophysiology. Topical corticosteroids (TCSs) are the mainstay therapy, but steroid phobia is emerging due to potential side effects. We aimed to determine the short-term effect of clobetasone butyrate on patients with AD.

**Methods:**

This investigator-blinded, randomised, moisturiser-controlled study evaluated patients with stable mild-to-moderate AD. Clobetasone butyrate ointment plus aqueous cream (Aq) or Aq alone was applied on randomised sites twice daily for 6 weeks. The itch score, modified Eczema Area and Severity Index (M-EASI) and epidermal biophysical parameters were assessed at baseline and 1 h, 3 h, 2 weeks and 6 weeks after application.

**Results:**

Sixteen patients, among whom 14 (87.5%) were women and two (12.5%) were men, participated in the study. There were no significant differences in pH, transepidermal water loss (TEWL) and hydration between TCS + Aq and Aq from 1 h to 6 weeks. A non-significant trend of pH increment was observed with TCS + Aq from baseline to 6 weeks. TEWL and hydration improved at 6 weeks for both treatment arms. The difference in TEWL from baseline was significant with Aq (P=0.01). The M-EASI at 6 weeks was comparable between the two arms. TCS + Aq improved itch and erythema better than Aq (P=0.02). No cutaneous adverse effects were observed at both sites.

**Conclusion:**

Short-term application of clobetasone butyrate with Aq is safe with no significant changes in epidermal biophysical parameters while controlling the symptoms and signs of eczema faster than Aq alone.

## Introduction

Atopic dermatitis (AD) is a chronic pruritic inflammatory skin disease that commonly occurs in children. Its prevalence is as high as 20% and continues to rise in both developed and developing countries.^1^ The epidermis of patients with AD is characterised by skin barrier dysfunction with increased transepidermal water loss (TEWL), less hydration and a surface pH that shifts towards alkalinity.^2^ The magnitude of these dysfunctions correlates with the severity of the disease.^3^

Topical therapy is the mainstay management for AD. Topical corticosteroids (TCSs) are the recommended first-line anti-inflammatory agents in both children and adults.^4^ Local cutaneous adverse effects of TCSs after prolonged use are well established. These effects include skin atrophy, hypopigmentation, telangiectasia, ecchymoses, striae and hypertrichosis. Adverse effects due to systemic absorption of TCSs are rare. Tachyphylaxis may occur with TCS usage, while withdrawal of TCS therapy may lead to a rebound of AD symptoms.^5^ Concurrent use of topical skin barrier repair agents is advisable to reduce TCS requirements and counter the adverse effects of TCSs on the stratum corneum.^6,7^ The adverse effects of TCSs have led to steroid phobia among patients with AD and their caretakers, resulting in compliance issues leading to inadequate therapy and disease control.^8,9^

Despite the established and well-recognised long-term adverse effects of TCSs, information on the short-term effects is sparse.^10,11^ This study aimed to assess the short-term effects of a moderate-potency TCS on the epidermal biophysical characteristics of patients with AD, specifically pH, TEWL, hydration and resolution of AD symptoms and signs.

## Methods

We performed a 6-week randomised controlled study at a dermatology clinic in a teaching hospital. Patients who were diagnosed with AD according to the Hanifin-Rajka criteria,^12^ were aged 7-40 years, had mild-to-moderate disease severity based on a Eczema Area and Severity Index (EASI)^13^ ranging from 1 to 21, had visible subacute or chronic lesions at symmetrical sites with no active skin infection or history of frequent infections and had been using TCSs either regularly or as needed and aqueous cream (Aq) with or without glycerine as a moisturiser were included in this study. Patients who were on systemic immunosuppressive or immunomodulating agents, were receiving phototherapy or had modifications in their treatment regimen within 4 weeks of recruitment were excluded from the study. Informed consent was obtained from patients or guardians of patients aged <18 years.

Demographic and clinical characteristics were obtained through face-to-face interviews and patients’ medical records. Physical examination was performed on all patients. The EASI was used to assess the overall AD severity.^13^

One eczematous lesion measuring 2-5 cm^2^ on the left and another on the right were identified for intervention and assessment; they were symmetrical lesions on either the upper limbs, lower limbs or abdomen. The lesion selected for TCS application was randomised using the sealed opaque envelope technique and treated with clobetasone butyrate ointment (U-Closone by HOE Pharmaceuticals) twice a day for 6 weeks. Clobetasone butyrate ointment is classified as a moderate-potency TCS in the Monthly Index of Medical Specialties and is the most commonly used moderate-strength TCS in our patients with AD. The lesion on the other side was not treated with any TCS. Both sides were moisturised twice a day using a standard Aq manufactured by KCK Pharmaceutical Industries with a pH ranging from 7.32 to 7.58 and without sodium laureth sulfate content. The investigators were blinded to the allocation of therapy. A moisturiser was applied on both sides to conform with standard clinical practice. Patients were instructed to apply the TCS about half an hour after the application of the moisturiser. They were allowed to continue their routine AD treatments, including TCS or topical calcineurin inhibitor (TCI) in other areas. Only two eczematous patches per patient were subjected to the intervention in this study to prevent flares of AD if large areas are denied TCS treatment, similar to the methodology of a few previous studies.^14-17^

The severity of eczema at the chosen sites was assessed using the M-EASI, whereby the total score was calculated without the inclusion of the body surface area. The itch score was measured based on lesional itch rather than overall itch using a visual analogue scale. The itch score, M-EASI, skin pH, TEWL and hydration were evaluated at baseline and 1 and 3 h after TCS application. These measurements were repeated after 2 and 6 weeks of regular TCS application. pH was measured using the HI99181 Skin pH Meter by Hanna Instruments, Padua, Veneto: Italy while TEWL was measured using Tewameter® TM300, Courage+Khazaka electronic GmbH, Köln: Germany. DermaLab Combo, Cortex Technology, Aalborg: Denmark was used to assess hydration. Patients were advised not to use any skin cleanser or apply any products on their skin for at least 12 h before the skin measurements and were acclimatised to the room environment for at least 15 min.

The sample size was calculated using the PS Power and Sample Size Calculation software version 3.1.6 based on the results of a previous study that compared the effect between betamethasone dipropionate and a vehicle on TEWL.^18^ The response within each group in this study was normally distributed with a standard deviation of 2.3, and the difference in the experimental and control means was 2.2. The estimated sample size was 18 per arm to be able to reject the null hypothesis that the means of the experimental and control groups are equal, with a probability (power) of 0.8 and a type I error probability of 0.05. Data were tabulated and analysed using the IBM® Statistical Package for the Social Sciences Statistics for Windows version 27.0, Armonk, NY: USA. Categorical data were presented as numbers and percentages. The Shapiro–Wilk test was utilised to determine the normality of continuous data. Continuous data were described as medians with interquartile ranges, as most were not normally distributed. The Mann–Whitney U test was used to analyse paired numerical data where applicable. A P-value of <0.05 was considered statistically significant.

## Results

A total of 16 patients with AD, including 14 (87.5%) women and two (12.5%) men, participated in the study. The median age at AD onset was 15 (22) years, and the median disease duration was 5.5 (20) years. The median EASI was 2.10 (2.66). Thirteen (81.3%) patients had a family history of atopy, and 13 (81.3%) had other atopic diseases, with the most common being allergic rhinitis (n=10, 12.82%). Five (31.3%) patients had a food allergy, while three (18.8%) had a drug allergy.

Seven (43.8%) patients were using perfumed liquid skin cleansers; five (31.3%), syndet cleansers; and four (25%), anti-septic cleansers. All patients used moisturisers, with 10 (62.5%) using humectant types; three (18.8%), emollient types; two (12.5%), occlusive types; and one (6.3%), other types. Fourteen (87.5%) patients were on TCSs, while two (12.5%) were not on any topical anti-inflammatory agents. Three (18.8%) patients had a history of systemic corticosteroid use. Thirteen (81.3%) patients were taking anti-histamines. The demographic, clinical and treatment characteristics of the study population are summarised in Table 1.

**Table 1:** Demographic, clinical and treatment characteristics.

Characteristics	N-16 n (%) or median (IQR)
Age at AD onset	15(2 2)
Duration of AD (year)	5.5 (20)
Family history of atopy	13(81.3%)
**Other personal atopic diseases**	
Allergic rhinitis	10 (62.5%)
Allergic conjunctivitis	3 (18.8%)
Urticaria	5(31.3%)
Bronchial asthma	4 (25%)
Food allergy	5(31.3%)
**AD disease severity (EASI)**	
Total score	2.10(2.66)
Almost dear	1 (6.3%)
Mild	12(75%)
Moderate	3(18.8%)
Severe	-
**Skin cleanser type**	
Perfumed liquid	7 (43.8%)
Syndet liquid	5(31.3%)
Anti-septic liquid	4 (25.0%)
**Moisturiser type**	
Occlusive	2 (12.5%)
Humectant	10 (62.5%)
Emollient	3(18.8%)
Others	1 (6.3%)
**Topical treatment**	
Corticosteroids	14 (87.5%)
Calrinetirin inhibitors	1
**Anti-histamine use**	13(81.3%)

IQR - interquartile range, EASI - Eczema Area and Severity Index.

### Effects of the TCS on the epidermal biophysical parameters

There were no significant differences in pH, TEWL and hydration at baseline between the TCS + Aq and Aq sites. No significant differences in all biophysical parameters were also observed between the two sites from 1 h to 6 weeks of application (Tables 2 and 3).

**Table 2:** Comparison of the changes in the biophysical parameters from baseline to 1 and 3 h after application at the TCS + Aq and Aq sites.

Parameters	Baseline			1 h			3 h		
	TCS + Aq Median (IQR)	Aq Median (IQR)	P-value	TCS + Aq Median (IQR)	Aq Median (IQR)	P-value	TCS + Aq Median (IQR)	Aq Median (IQR)	P-value
**pH**	5.11 (1.17)	5.27 (1.07)	0.90	Δ +0.12 (0.22)	Δ +0.01 (0.35)	0.20	Δ +0.08 (0.24)	Δ -0.02 (0.34)	0.29
**TEWL**	12.06 (10.60)	12.93 (5.29)	1.00	Δ +0.24 (5.15)	Δ -0.63 (5.91)	0.75	Δ +0.32 (5.40)	Δ +0.07 (6.86)	0.22
**Hydration**	193.50 (192.00)	165.50 (123.00)	0.75	Δ -3.00 (99.00)	Δ -12.5 (83.00)	0.96	Δ -31.00 (84.00)	Δ -29.50 (85.00)	0.84

Δ, changes in the values compared with the baseline. IQR, interquartile range. TEWL, transepidermal water loss. TCS, topical corticosteroid. Aq, aqueous cream.

**Table 3:** Comparison of the changes in the biophysical parameters from baseline to 2 and 6 weeks after application at the TCS + Aq and Aq sites.

Parameters	Baseline		2 weeks			6 weeks		
	TCS + Aq Median (IQR)	Aq Median (IQR)	TCS + Aq Median (IQR)	Aq Median (IQR)	P-value	TCS + Aq Median (IQR)	Aq Median (IQR)	P-value
**pH**	5.11 (1.17)	5.27 (1.07)	Δ 0.07 (0.51)	Δ 0.07 (0.40)	0.83	Δ 0.24 (0.62)	Δ 0.16 (0.82)	0.56
**TEWL**	12.06 (10.60)	12.93 (5.29)	Δ -2.54 (9.87)	Δ -2.15 (9.25)	0.92	Δ -1.33 (8.05)	Δ -4.13 (8.52)	0.92
**Hydration**	193.50 (192.00)	165.50 (123.00)	Δ -21.50 (129.00)	Δ 20 (139.00)	0.78	Δ 20.5 (111.00)	Δ -11 (121.00)	0.56

A, changes in the values compared with the baseline. IQR, interquartile range. TEWL, transepidermal water loss. TCS, topical corticosteroid. Aq, aqueous cream.

A non-significant trend of increment in pH from baseline to 6 weeks was observed at the TCS + Aq sites (Table 4). TEWL showed a progressive reduction from baseline to 6 weeks, which was also non-significant. Hydration initially decreased but was greater than the baseline by 6 weeks (P=0.38) (Table 4). The pH at the Aq sites was slightly lower than the baseline at 6 weeks (P=0.85), while TEWL showed a gradual reduction until 6 weeks (P=0.01). Similar to hydration at the TCS + Aq sites, the values initially decreased but became higher than the baseline by 6 weeks at the Aq sites (P=0.72) (Table 4).

**Table 4:** Biophysical parameters at the TCS + Aq and Aq sites from baseline to 6 weeks after application.

Parameters	Baseline Median (IQR)	1 h Median (IQR)	P-value (baseline vs 1 h)	3 h Median (IQR)	P-value (baseline vs 3 h)	2 weeks Median (IQR)	P-value (baseline vs 2 weeks)	6 weeks Median (IQR)	P-value (baseline vs 6 weeks)
**TCS + Aq**									
pH	5.11 (1.17)	5.41 (0.87)	0.12	5.19 (1.19)	0.89	5.25 (0.56)	0.63	5.36 (0.64)	0.30
TEWL	12.06 (10.6)	11.67 (9.68)	0.87	11.75 (10.85)	0.40	9.54 (11.72)	0.46	6.19 (13.04)	0.20
Hydration	193.5 (192)	178 (77)	0.31	174.5 (137)	0.26	157 (137)	0.67	226.5 (151)	0.38
**Aq**									
pH	5.27 (1.07)	5.31 (0.83)	0.62	5.13 (0.99)	0.28	5.30 (0.60)	0.94	5.19 (0.67)	0.85
TEWL	12.93 (5.29)	10.63 (19.57)	0.29	9.94 (9.66)	0.14	9.82 (7.17)	0.90	6.23 (6.49)	**0.01**
Hydration	165.5 (123)	168 (100)	0.61	160 (94)	0.29	184(102)	0.97	203 (183)	0.72

IQR, interquartile range. TEWL, transepidermal water loss. TCS, topical corticosteroid. Aq, aqueous cream.

### Effects of the TCS on the itch score and lesional severity

The redness score at the TCS + Aq sites showed an earlier reduction at 2 weeks (median=0 [1]) than that at the Aq sites (median=1 [0]) (P=0.002). At 6 weeks, both sites achieved resolution of redness. Thickness and scratching resolved within 2 weeks at both sites. No significant lichenification was seen among the patients. The TCS + Aq sites showed better improvement at 2 weeks, with an itch score of 0.5 (2), than the Aq sites, with an itch score of 1.5 (2); however, the difference was not statistically significant. Itching resolved at both sites by 6 weeks. These findings are summarised in Table 5. Skin atrophy, hypopigmentation, telangiectasia, hypertrichosis and folliculitis were not observed at the TCS application sites in all patients.

**Table 5:** Itch score and disease severity.

Parameters	Baseline			2 weeks			6 weeks		
	TCS + Aq Median (IQR)	Aq Median (IQR)	P-value	TCS + Aq Median (IQR)	Aq Median (IQR)	P-value	TCS + Aq Median (IQR)	Aq Median (IQR)	P-value
**Itch score (0–10)**	3.5 (3)	3.5 (3)	1.00	0.5 (2)	1.5 (2)	0.10	0 (0)	0 (2)	0.02
**M-EASI, total**	3 (3)	3 (3)	0.84	0 (2)	1 (2)	0.07	0 (0)	0 (1)	0.21
Redness	2 (1)	1.5 (1)	0.78	0 (1)	1 (0)	0.03	0 (0)	0 (1)	0.38
Thickness	0 (1)	0 (1)	1.00	0 (0)	0 (1)	0.14	0 (0)	0 (0)	0.78
Scratching	1 (1)	1 (1)	1.00	0 (0)	0 (0)	0.56	0 (0)	0 (0)	0.78
Lichenification	0 (0)	0 (0)	-	0 (0)	0 (0)	-	0 (0)	0 (0)	-

M-EASI, modified Eczema Area and Severity Index. TCS, topical corticosteroid. Aq, aqueous cream

## Discussion

TCSs are the mainstay topical agents for treating AD, which are highly effective in controlling cutaneous inflammation. The various types of TCSs are classified based on their potency. The occurrence of adverse effects depends on the type, potency, duration of use and site of application of TCSs. Subclinical adverse effects can be measured using various parameters including epidermal thickness, stratum corneum adhesion and integrity, surface pH, TEWL and hydration. Conflicting results have been reported for the adverse effects of shortterm use of a few potent TCSs.^14-16^ Epidermal thinning may occur within 10 days to 4 weeks of regular application of betamethasone valerate and clobetasol propionate.^15,18,19^ Decreased stratum corneum adhesion and integrity have been demonstrated as early as 3 days with clobetasol 0.05%, a potent TCS.^14^ However, improved TEWL and hydration and increased lamellar bodies were observed with the 3-week use of triamcinolone acetonide in a previous study,^15^ while mometasone furoate increased ceramide levels after 12 weeks with no change in TEWL and hydration in another study.^16^ Another report indicated that the application of betamethasone valerate 0.1% cream twice per week for 8 weeks in patients with quiescent AD resulted in increased pH, unchanged TEWL and loss of stratum corneum cohesion but preserved stratum corneum integrity.^17^ Most of these studies did not fully reflect the use of standard topical therapies in clinical practice, as moisturisers were not used concurrently with TCSs.^14-19^

The skin’s acid mantle is important in maintaining its normal structure and functions. The physiologic pH of the stratum corneum ranges from 4.1 to 5.8 with small variations between the face, trunk and extremities.^20^ pH is increased by 0.2-0.3 units in AD lesions compared with non-lesional skin and by 0.2 units in non-lesional AD skin compared with healthy controls.^21^ Moisturisers with a physiological pH have been shown to improve skin barrier function compared with standard moisturisers with a higher pH, while skin cleansers with a low pH have been reported to improve disease severity in patients with AD.^22,23^ Maintaining the skin pH on the acidic spectrum is beneficial in controlling AD. In the present study, we demonstrated that short-term use of clobetasone butyrate ointment with a standard Aq with a pH ranging from 7.32 to 7.58 did not alter the skin pH of our study population. Although a topical agent that reduces pH to physiological levels is more desirable, a TCS that does not alter pH is acceptable.

TEWL is a component of the skin barrier that is markedly affected in AD. It is correlated with disease severity and gradually declines with clinical improvement.^2,3^ The effects of TCSs on TEWL in patients with AD have been documented in a few studies. TEWL increment has been observed in atrophic skin following long-term use of highly potent TCSs.^10,11^ Shortterm application of clobetasol propionate, a potent TCS, has increased TEWL, reduced dermal and epidermal thickness and decreased the levels of epidermal ceramides, cholesterol and free fatty acids, which are important for maintaining epidermal integrity.^24^ Conversely, concurrent application of betamethasone valerate 0.1%, another potent TCS, with a moisturiser for 3 weeks has improved TEWL and hydration.^15^ However, epidermal thickness has been reduced, and the structures of lamellar bodies and the lipid bilayer have not been restored.^15^ Mometasone fUroate cream, another potent TCS, used in combination with a moisturiser has increased ceramide levels and has not affected TEWL and hydration after 12 weeks.^16^ A study on triamcinolone acetonide cream, a moderate-potency TCS, showed improved TEWL and hydration and a larger number of physiological lamellar bodies after 3 weeks.^15^ In our study, the use of a moderate-potency TCS with a moisturiser did not negatively impact TEWL but improved hydration. Moderate-potency steroids may exert a different effect on epidermal characteristics compared with potent steroids; as demonstrated by previous reports and the present findings, concomitant use of moisturisers may also play a role in influencing the effects of TCSs. Topical anti-inflammatory treatment for AD in combination with moisturisers has been shown to yield better results than monotherapy.^25^ Moisturisers work synergistically with TCSs by enhancing their effectiveness and playing a steroid-sparing role.^4,25^ Moisturisers prolong the time between AD flares, decrease the frequency of flares and reduce the amount of TCSs required for disease control.^25^ TCSs provide faster resolution of inflammation, while moisturisers repair and preserve the epidermal barrier. In our study, both arms achieved good symptom control, with TCS application demonstrating faster resolution of dermatitis. TCS application was more effective than moisturiser application alone in lesion clearance. The beneficial effect of using moisturisers even as a monotherapy was observed.

In summary, short-term moderate-potency TCS application in patients with mild-to-moderate AD did not affect the epidermal barrier parameters nor did it yield local cutaneous adverse effects. TCS application resulted in faster recovery of the affected skin than moisturiser application alone. These findings can be used to reassure patients and caregivers, especially those with steroid phobia, that shortterm use of TCSs to control AD would not impair the skin barrier functions while achieving better symptom control and may improve their compliance in using moisturisers.

In our study, the patients were advised to comply with the study requirements including the topical applications; however, compliance was not objectively measured, which may be a limitation of this study.

## Conclusion

Short-term application of a moderate-potency TCS with Aq for mild-to-moderate AD does not significantly change skin pH, TEWL and hydration while providing rapid relief of the symptoms and signs of eczema. These findings can reassure patients with AD that short-term use of TCSs is safe. As moisturiser monotherapy also improves eczema, moisturisers should be used with TCSs to optimise treatment benefits and limit the adverse effects of TCSs.

## Data sharing statement

Data are available upon request.

**How does this paper make a difference in general practice?**

- Moderate-potency topical steroids are safe for short-term use.
- Transepidermal water loss and epidermal hydration improve, and no adverse effects are observed clinically after 6 weeks.
- Topical steroids should be used in combination with moisturisers.
- Moisturisers alone are adequate in treating mild eczema, but topical corticosteroids can hasten the resolution of itching and erythema.
